# Supplementary material for: Unhealthy Food at Your Fingertips: Cross-Sectional Analysis of the Nutritional Quality of Restaurants and Takeaway Outlets on an Online Food Delivery Platform in New Zealand
Source: Nutrients. 2022 Oct 30;14(21):4567. doi: 10.3390/nu14214567 (PMC9656530; doi:10.3390/nu14214567)
Supplement: Supplementary file 1 [file nutrients-14-04567-s001.zip › nutrients-1941858-supplementary.pdf]

Supplementary Table S1. Proportion of Discretionary and FFG for Combination menu items on Uber Eats. The proportion of food and beverage categories as part of combination menu items in popularized (N = 442 unique combination menu items) and regular (n = 3304 unique combination menu items) menu items from 305 unique local independent and franchise takeaway outlets. Regular menu items exclude popularized menu items.

Type of Category	Food Categories	Popularized Menu Items		Regular Menu Items	
		n	%	n	%
Discretionary	Fried Potato (or similar)	289	65.4	1752	53.0
	Cereal-based mixed meal	226	51.1	1577	47.7
	Meat or alternative based mixed meal	151	34.2	1298	39.3
	Sugar Sweetened Beverages	79	17.9	543	16.4
	Baked goods/Desserts (homemade or similar)	87	19.7	159	4.8
	Other Beverage[b]	110	24.9	170	5.1
	Vegetable-based mixed meal	39	8.8	347	10.5
	Discretionary Milk Based Beverages	0	0	0	0
	Savoury Sauces, Condiments and Spreads	117	26.5	893	27.0
	Iced confectionary and dairy-based desserts	76	17.2	157	4.8
	Other Food[a]	2	0.5	45	1.4
	**Total Discretionary (n)**	**1176**	**90.0**	**6941**	**81.1**
Five Food Groups (FFG)	Cereal-based mixed meal	9	2.0	202	6.1
	Meat or alternative based mixed meal	24	5.4	131	4.0
	Other Beverage[d]	0	0	18	0.5
	Vegetable-based mixed meal	34	7.7	130	3.9
	Other Food[c]	61	13.8	609	18.4
	Water	2	0.5	429	13.0
	Juice	0	0	102	3.1
	**Total FFG (n)**	**130**	**10.0**	**1621**	**18.9**
	**Total (n)**	**1306**		**8562**	
	**Total Unique Combination menu items**	**442**		**3304**	

[a]Confectionery, Discretionary snack food (Savoury) – Packaged, Discretionary snack food (Sweet) – Packaged, Other snack food (other), Processed Meats, [b]Alcohol, Energy Drinks, Non-Sugar Sweetened Beverages, Rehydration Beverages (Electrolytes), Water Based Flavoured Beverage – sugar not determined, [c]Breads and Cereals, Dairy and alternatives, Fats/Oils, Fruit, Legumes, Meat and Alternatives, Soup, Vegetables, Vegetables (Other), [d]Body Building and Performance Beverages, Coffee, Milk/Milk Alternatives, Milk/Milk Alternative Based Beverages, Tea. % The percentages are out of total unique combination meals.
